# Raman Spectroscopy and Microscopy Applications in Cardiovascular Diseases: From Molecules to Organs

**DOI:** 10.3390/bios8040107

**Published:** 2018-11-12

**Authors:** Ardalan Chaichi, Alisha Prasad, Manas Ranjan Gartia

**Affiliations:** Department of Mechanical and Industrial Engineering, Louisiana State University, Baton Rouge, LA 70803, USA; achaic1@lsu.edu (A.C.); aprasa9@lsu.edu (A.P.)

**Keywords:** Raman imaging, vibrational spectroscopy, cardiovascular disease, cardiac hypertrophy, cardiac biomarkers

## Abstract

Noninvasive and label-free vibrational spectroscopy and microscopy methods have shown great potential for clinical diagnosis applications. Raman spectroscopy is based on inelastic light scattering due to rotational and vibrational modes of molecular bonds. It has been shown that Raman spectra provide chemical signatures of changes in biological tissues in different diseases, and this technique can be employed in label-free monitoring and clinical diagnosis of several diseases, including cardiovascular studies. However, there are very few literature reviews available to summarize the state of art and future applications of Raman spectroscopy in cardiovascular diseases, particularly cardiac hypertrophy. In addition to conventional clinical approaches such as electrocardiography (ECG), echocardiogram (cardiac ultrasound), positron emission tomography (PET), cardiac computed tomography (CT), and single photon emission computed tomography (SPECT), applications of vibrational spectroscopy and microscopy will provide invaluable information useful for the prevention, diagnosis, and treatment of cardiovascular diseases. Various in vivo and ex vivo investigations can potentially be performed using Raman imaging to study and distinguish pathological and physiological cardiac hypertrophies and understand the mechanisms of other cardiac diseases. Here, we have reviewed the recent literature on Raman spectroscopy to study cardiovascular diseases covering investigations on the molecular, cellular, tissue, and organ level.

## 1. Introduction

**Figure 1:**
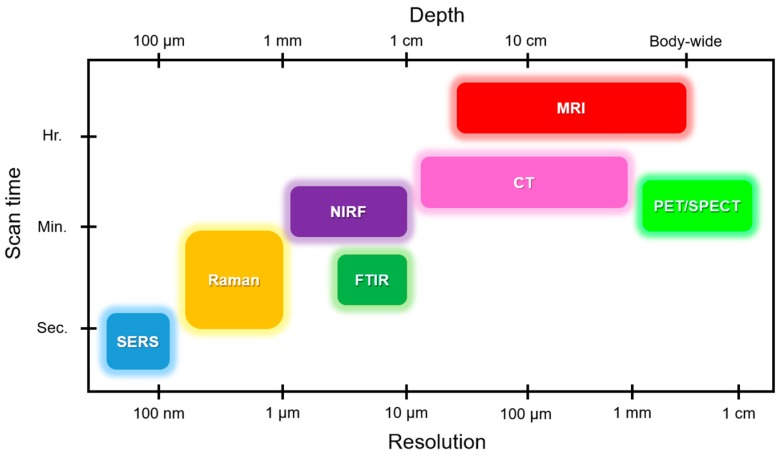
Comparison of various methods for cardiovascular studies in terms of analysis depth, resolution, and scan time. Raman and surface enhanced Raman spectroscopy (SERS) provide the highest resolution and shortest scan time. However, conventional methods like MRI and CT show the greatest depth of analysis. FTIR, Fourier-transform infrared spectroscopy; MRI, magnetic resonance imaging; CT, computed tomography; PET, positron emission tomography; SPECT, single-photon emission computerized tomography; NIRF, near infrared fluorescence.

Recent advances in vibrational spectroscopy and microscopy have facilitated the use of this approach for biomedical applications. Two major applications of Raman spectroscopy techniques in clinical use are the diagnosis of certain medical condition and the quantification of analytes [1,2,3,4,5]. The limit of penetration depth for performing such analyses in vivo is usually on the order of millimeters [6]. However, some studies have reported achieving several centimeters of effective depth by utilizing transmission Raman spectroscopy [7] and spatially offset Raman spectroscopy [8] methods. These techniques of probing deeper tissues have been achieved by optimizing the properties of photon diffusion in opaque media like some conventional approaches (fluorescence tomography and near infrared absorption). Meanwhile, Raman spectroscopy is naturally a label-free method, and has significantly greater chemical specificity and spatial imaging resolution compared to other techniques (Figure 1) [1,9,10]. The use of time-gated approaches in Raman spectroscopy such as video-rate coherent anti-Stokes Raman scattering spectroscopy (CARS) and stimulated Raman scattering (SRS) is mostly responsible for the current excitement over achieving deep tissue analysis [11,12]. However, the unreasonable expense and complexity of such methods hinder their practical use.

Although most of the clinical studies on Raman spectroscopy focus on spectral characteristics of the fingerprint region (<1800 cm^−1^) [13], distinctive features of numerous proteins and lipids appear at higher wavenumbers (2200 to 4500 cm^−1^) [14]. Therefore, high wavenumber Raman spectroscopy is also a promising tool for in vivo studies of various diseases when fingerprint range spectral analysis fails. Cervical cancer [15,16,17], skin cancer [18,19,20], gastrointestinal tract disorders [21], brain disorders [22], and cardiovascular diseases [23] are the most attractive clinical applications of Raman spectroscopy. As they are responsible for approximately 30% of adult deaths, cardiovascular diseases pose an even greater danger than cancer in terms of mortality rate [24]. Diagnosis has been always considered the most challenging part of cardiac pathology [25]. Increased myoglobin (Mb) is the most common indicator of myocardial damage [24]. Therefore, heart attack or acute myocardial infarction (AMI) is ubiquitously diagnosed by detection of Mb level in plasma serum. Mb levels increase up to 3–6 times higher than normal values (5–25 IU/L) in plasma serum after about 5 h of AMI occurrence [26]. Although quick detection of AMI is crucial to minimize the fatal consequences of such an attack, current Mb detection methods are still considerably slow and unaffordable. Accordingly, various Raman-based sensing, spectroscopy, and microscopy methods can be used in cardiac disease management. Most of these methods are cost-efficient, rapid, and practical for use in clinical applications. For instance, surface enhanced Raman spectroscopy (SERS) has proved to be a promising approach for ultra-low concentration (10^−15^ mol/L) detection of biochemicals and analytes by means of a disposable sensor [27].

In addition to molecular- and cellular-level investigation, Raman spectroscopy can also be utilized to probe tissue-level anomalies such as cardiac hypertrophy, which is abnormal thickening and enlargement of the heart muscle [28]. This is usually caused by extraordinary enlargement of cardiac muscle cells, known as cardiomyocytes. This might originate from valvular disease and hypertension (pathological hypertrophy) or just be due to regular exercise in athletes (physiological hypertrophy) [29]. Physiological hypertrophy does not generally result in heart failure. However, pathological hypertrophy is considered dangerous and might cause serious injury to cardiac muscle cells [30]. Therefore, a thorough comprehension of cardiac hypertrophy can dramatically mitigate the risk factors of heart failure. Although there is still no established method for differentiation of pathological and physiological hypertrophies, distinguishing the aforementioned hypertrophy categories seems possible by Raman spectroscopy and imaging techniques. By finding the signatures of enlarged cardiac cells for both types of hypertrophy, either in the fingerprint region or higher wavenumbers, it is possible to study the mechanism of cardiac hypertrophy. Moreover, Raman-based cardiac endoscopy methods can be utilized for diagnosis and treatment purposes (Table 1). Although various Raman spectroscopy techniques have great potential to investigate cardiac diseases, there are still not enough studies in this field. In this review, current and potential applications of Raman spectroscopy for cardiac studies are elaborated to emphasize the significance of this field.

## 2. Raman Scattering Applications for Cardiac Studies

### 2.1. Principle of Raman Scattering

Raman scattering is an inelastic light scattering phenomenon. When a monochromatic light is incident on a sample, the incident light is scattered both elastically and inelastically. The elastically scattered light (also known as the Rayleigh effect) returns with the same energy as incident light, while the inelastic scattered light (also known as the Raman effect) returns with a different wavelength. This difference corresponds to an energy shift termed the Raman shift, which provides unique fingerprints of the molecules [49].

**Figure 2:**
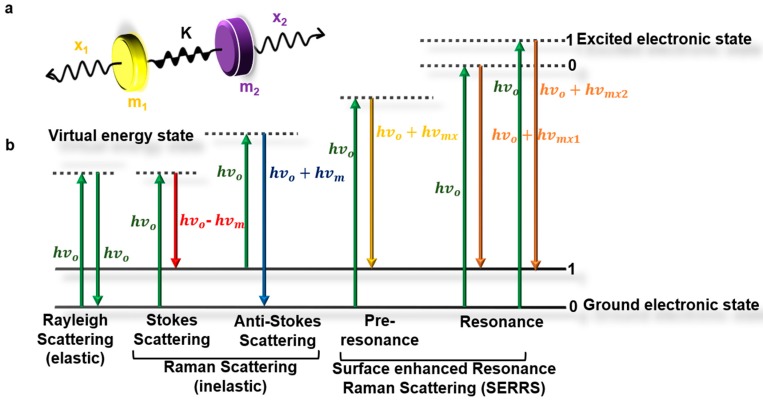
Theory of Raman effect. (**a**) Interpretation of Raman scattering via displacement of two diatomic molecules suspended on a spring. (**b**) Jablonski diagram showing transition of energy for Rayleigh and Raman scattering.

Mathematically, the theory of Raman effect can be explained considering two diatomic molecules with mass *m*_1_ and *m*_2_ on a spring with bond strength *K* and displacement *x*, as shown in Figure 2a. The classical model of the displacement of the molecules can be described by Hooke’s law [50] as shown in Equation (1):

(1) $$\frac{m_{1}m_{2}}{m_{1}+m_{2}}\left(\frac{d^{2}x_{1}}{dt^{2}}+\frac{d^{2}x_{2}}{dt^{2}}\right)=-K\left(x_{1}+x_{2}\right)$$

Simplifying Equation (1) by substituting $\frac{m_{1}m_{2}}{m_{1}+m_{2}}$ by *µ* and $\left(x_{1}+x_{2}\right)$ by *q*, Equation (1) becomes:

(2) $$\mu \frac{d^{2}q}{dt^{2}}=-Kq$$

In terms of *q*, Equation (2) becomes:

(3) $$q=q_{o}\cos\left(2\pi v_{m}t\right)$$

in which the molecular vibration (*ν_m_*) is:

(4) $$v_{m}=\frac{1}{2\pi }\sqrt{\frac{K}{\mu }}$$

Equations (3) and (4) show that molecular vibrations follow a cosine function frequency that is dependent on *K* (bond strength) and *µ* (reduced mass), resulting in each molecule having a unique vibrational signature. The vibrational frequencies can be quantified, since molecular polarizability is a function of displacement. Therefore, due to the interaction of light and molecule, a dipole moment *P* is induced, which is a result of molecular polarizability ⍶ and electric field *E*_0_, as shown in Equation (5):

(5) $$P=\underset{¯}{\alpha }E_{0}\cos\left(2\pi v_{0}t\right)$$

The vibrational frequency *v*_0_ can be deduced by combining polarizability in Equation (5) with Equation (3) as a linear function of displacement, as shown in Equation (6):

(6) $$P=\underset{¯}{\alpha }E_{0}\cos\left(2\pi v_{0}t\right)+q_{0}\cos\left(2\pi v_{m}t\right)\;E_{0}\cos\left(2\pi v_{0}t\right){\left[\frac{d\underset{¯}{\alpha }}{dt}\right]}_{q=0}$$

The two parts of Equation (6) confirm that the incident light can be described as scattered light with two components: (i) Rayleigh scatter, in which the frequency of incident light is constant, and (ii) Raman scatter, which results in a shift in frequency of incident light. This shift can be either an increase (anti-Stokes shift) or decrease (Stokes shift) in frequency, as shown in Equation (7) (Figure 2b) by expanding Equation (6) [50]:

(7) $$q_{0}\;E_{0}{\left[\frac{d\underset{¯}{\alpha }}{dt}\right]}_{q=0}\;\left[\cos\left(2\pi \{v_{0}+v_{m}\}t\right)+\cos\left(2\pi \{v_{0}-v_{m}\}t\right)\right]$$

According to the modern theories of Raman spectroscopy, incident radiation is considered as a nondivergent and monochromatic beam, and ω is defined as the angular frequency. Furthermore, it is assumed that the molecule is fixed at zero of X, Y, and Z in Cartesian coordinates. Moreover, the incident radiation wavelength $\left(\bar{E}\right)$ is considered to be dramatically larger than the molecule size. It should be also assumed that the beam incident and polarization directions are on the Z and Y axis, respectively. According to these assumptions, polarization square amplitude ($\bar{\mu^{ind}{}_{fi}^{2}}$) in the YZ plane can be defined as follows [51,52,53]:

(8) $$\bar{\mu^{ind}{}_{fi}^{2}}={\left[\alpha_{YY}^{fi}\left(\omega \right)E_{Y}^{\omega }e^{-i\omega t}+c.c.\right]}^{2}+{\left[\alpha_{ZY}^{fi}\left(\omega \right)E_{Y}^{\omega }e^{-i\omega t}+c.c.\right]}^{2}$$

in which $\alpha^{fi}\left(\omega \right)$, $E_{Y}^{\omega }$, and *fi* are defined as molecular polarizability, electric field amplitude, and initial/final transition moment, respectively. As a result, Raman intensity in the aforementioned conditions can be described as [51]:

(9) $$I_{s}=\frac{d\varphi }{d\Omega }=\frac{\omega_{s}^{4}\bar{\mu^{ind}{}_{fi}^{2}}}{32\pi^{2}\varepsilon_{0}c^{3}}=I_{0}\frac{d\sigma }{d\Omega }$$

in which dφ, dΩ, *c*, $\varepsilon_{0}$ are assumed to be radiation power, solid angle conical beam, light speed, and vacuum permittivity, respectively.

Determining the number of scattered photons (*N_s_*) with respect to angular frequency (*ω_s_*) is another approach to measure the intensity of scattered light by means of the following expression [51]:

(10) $$I_{s}=\frac{N_{s}\hbar \omega_{s}}{d\Omega }$$

where dΩ is constant and defined as a solid angle element in which the photons are scattered. Therefore, the number of scattered photons is dependent on the ratio $\frac{I_{s}}{\hbar \omega_{s}}$.

Researchers have applied these concepts and assembled them with common analytical tools such as a microscope and an endoscope for monitoring, imaging, and molecular fingerprinting. Examples of microscope- and endoscope-supported imaging include Raman spectroscopy, Raman microspectroscopy (RMS), surface enhanced Raman spectroscopy (SERS), and confocal Raman microscopy (either inverted or upright).

### 2.2. Raman Imaging Applications

**Figure 3:**
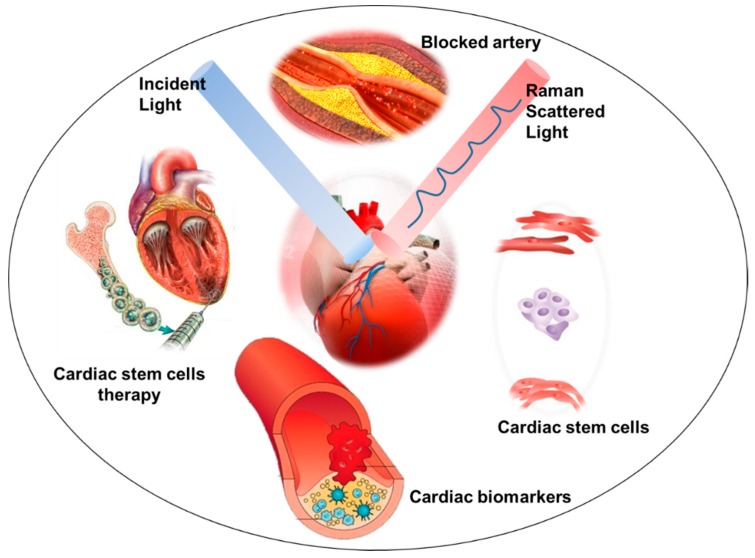
Schematic showing Raman spectroscopy use in cardiac applications.

**Figure 4:**
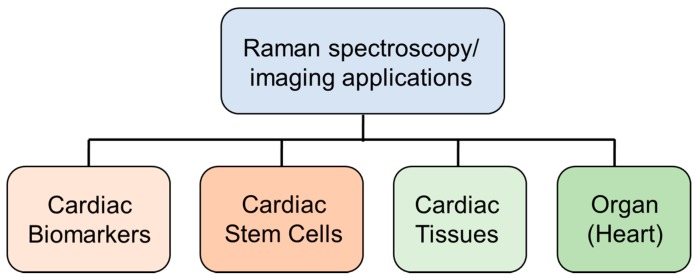
Schematic showing the organization of the review.

Since the human heart is a nonregenerative organ, inevitable events such as abnormal heart rhythms, cardiac arrest, or damage to cardiac tissue can be very risky to human health and life. In recent years, researchers have achieved considerable success in using cell-based therapies as an alternative to heart transplants to replace damaged cardiomyocytes [29,30]. Although they offer great promise, these techniques need to be improved in order to produce high cell populations with better procedures for optimal clinical outcomes at sustainable costs [54]. Advantages of Raman imaging are that it is a label-free approach, it allows measurement of samples in any state (liquid/solid), and it requires little to no sample preparation [55] (Figure 3). It is important for the characterization of biomaterials such as cells and tissues, as they can be investigated in their native state without adding further variability to the analysis. Furthermore, Raman spectroscopy is not affected by the presence of water/phosphate buffered saline (PBS), which is essential for cell/tissue analysis. Figure 4 shows the approach of this review for Raman spectroscopy and imaging techniques used in different biological applications.

#### 2.2.1. Raman Spectroscopy for Cardiac Biomarker Detection

##### Cardiac Biomarkers

During cardiac injury, generally three cardiac troponin complexes are released, cardiac troponin C (cTnC), cardiac troponin T (cTnT), and cardiac troponin I (cTnI). Among these, the concentration of cTnI in the serum after 3–4 h of stroke has been found to be very low, i.e., ~1–3 ng/mL, and peaks usually at 12–24 h [3]. Furthermore, the relative concentration of cTnI remains high for up to 3 to 5 days, and it starts to meet the normal state in 7 to 14 days (Table 2) [56]. To investigate this, several studies have been conducted to identify the cTnI cardiac biomarker in patients with myocardial infarction (MI) within this specified time span. In order to achieve detection at such low concentrations, scientists synthesized optical microspheres by depositing silver nanoparticles (AgNPs) on its surface to make optical resonators. cTnI molecules adsorbed onto the AgNPs via the dextran layer were detected in HEPES buffered solution (HBS). The coupling of optical microspheres with an optical fiber generates localized surface plasmon resonance (LSPR) near the AgNPs within the whispering gallery mode’s evanescent field. Accordingly, upon application of λ excitation = 565 nm, the AgNPs (d = 50 nm) were excited into plasmonic mode. Deep red indicates the highest intensity, and the local field enhancement around the AgNPs was mostly observed in hotspots. The LSPR enhanced the Raman signals, improving the structure sensitivity. Hotspots experienced the highest amount of enhancement, by representing a robust electromagnetic field (EF). The EF around the AgNPs exceeded 10^2^, resulting in Stokes Raman enhancement on the order of 10^10^ [31]. The Raman peaks for cTnI are predominantly within 1224 cm^−1^ to 1293 cm^−1^. cTnI also contains tyrosine (at 848 cm^−1^) and phenylalanine (at 1018 cm^−1^) residues [31].

**Figure 5:**
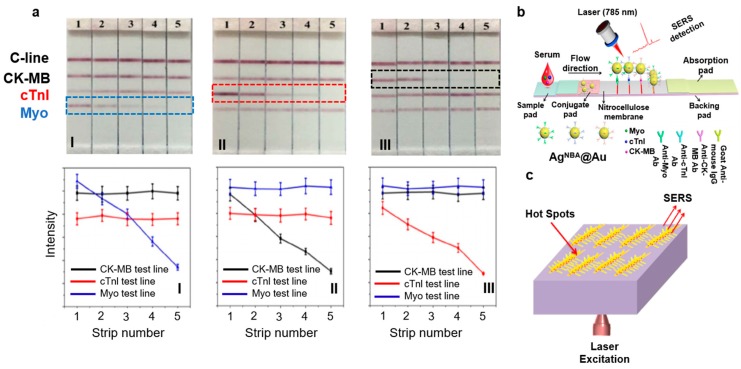
Cardiac biomarkers (Raman spectral signatures). (**a**) Pictures of SERS LFA strips (top) and their representative Raman intensity peaks (bottom) (excitation wavelength: 785 nm). (Reprinted with permission from [32].) (**b**) Schematic representation of core-shell SERS nanotag-based multiplex LFA (excitation wavelength: 785 nm). (Reprinted with permission from [32].) (**c**) Schematic representation of Ag NPT/ITO substrate for SERS-active surface for monitoring of myoglobin proteins (excitation wavelength: 785 and 485 nm). (Reprinted with permission from [23].)

Point-of-care testing (POCT) designed to acquire quick and cost-effective health information has gathered much attention recently. These stand-alone portable devices are based on the concept of interactive binding of an analyte of interest (for example, an antigen) with its counterproteins (for example, an antibody) tagged with a fluorophore to see visible color changes [57]. With the idea of expanding the reach of personalized diagnosis of MI, a proof-of-concept device intended for lateral flow assay (LFA) was demonstrated to detect three cardiac biomarkers quantitatively in a relatively short amount of time. The nitrocellulose (NC) strips for LFA were encapsulated with Raman dyes inside the Ag core and Au shell nanoparticles (NPs) in order to create SERS nanotags for detection of Myo, cTnI, and creatine kinase–muscle/brain (CK-MB) cardiac biomarkers. As shown in Figure 5a, four lines with a 3 mm gap representing C-line (reference line in red), and three test lines for CK-MB, cTnI, and Myo were arranged on the NC membrane. Figure 5a shows the following: The concentration in I was cTnI: 20 ng/mL; CK-MB: 60 ng/mL; and Myo 1–5: 200, 50, 10, 1, and 0.1 ng/mL, respectively. The concentration in II was cTnI 1–5: 50, 10, 3, 1, and 0.1 ng/mL, respectively; CK-MB: 60 ng/mL; and Myo: 100 ng/mL. The concentration in III was cTnI: 20 ng/mL; CK-MB 1–5: 60, 10, 1, 0.5, and 0.1 ng/mL, respectively; and Myo: 100 ng/mL. In I, cTnI and CK-MB displayed the same intensity of red irrespective of Myo concentration. The Myo test line became redder and its corresponding Raman spectrum intensity increased with concentration. A similar trend was seen in II and III, which indicated the absence of cross-reaction between the three biomarkers. Figure 5a (bottom) shows Raman peaks even at very low concentrations, indicating poor quantitative ability. In conclusion, POCT devices can only provide a semiquantitative yes/no response [32]. The schematic of a typical LFA is shown in Figure 5b.

SERS has also been utilized to detect biomarkers during MI. MI is one of the most common life-threating conditions worldwide. To detect MI, the World Health Organization (WHO) [23] has approved many cardiac biomarkers, among which myoglobin is found to be released into the bloodstream within ~1 h of occurrence of chest pain. A study reported increased levels of myoglobin from approximately 90 to 250 ng/mL in the bloodstream within 90 min after an episode of MI [10]. Gold (Au), silver (Ag), and copper (Cu) are well-known metals that amplify Raman signals. Many studies have been reported on utilizing these metals as nanoparticles (NPs), nanorods, nanowells, and nanopore arrays with large LSPR to serve as dynamic SERS substrates. Another advantage of using such nanoparticle surfaces is enhancement of Raman signal by an order of magnitude of 10^3^ to 10^6^ compared to normal Raman spectroscopy. In a recent study, a group of researchers [23] exploited the advantages of metal nanostructures and utilized a SERS myoglobin sensor. As shown in Figure 5c, the SERS coupled sensor comprised a 3D silver-based nano-Pinetree array (NPT) modified with indium tin oxide (ITO) to form an Ag NPT/ITO substrate. The limit of detection (LOD) of this sensor was found to be 10 ng/mL, which was comparatively lower than the physiological myoglobin level of ~250 ng/mL within 90 min of MI [23].

#### 2.2.2. Raman Spectroscopy for Cardiac Cells and Cardiac Stem Cells

**Figure 6:**
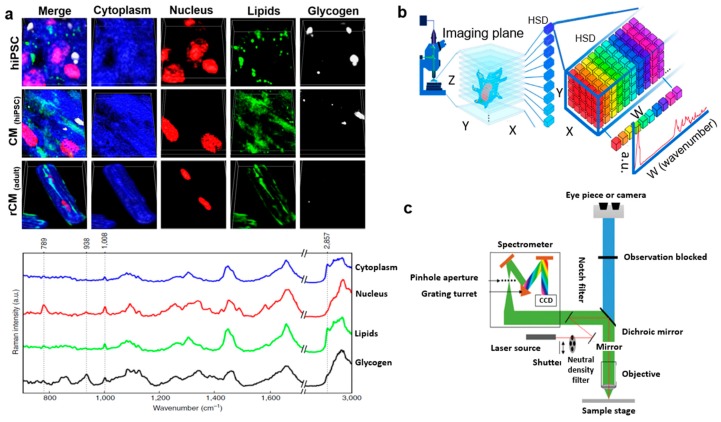
Raman imaging of cardiac cells. (**a**) 3D visualization of representative human-induced pluripotent stem cells, cardiomyocytes, and adult rat ventricular cardiomyocytes (top), and representative Raman spectra (bottom) (excitation wavelength: 532 nm). (Reprinted with permission from [33]). (**b**) Graphic illustration of quantitative volumetric Raman imaging process, data collection, spectral unmixing, and 3D reconstruction of stem cells (excitation wavelength: 532 nm). (Reprinted with permission from [33]). (**c**) Standard configuration of an upright confocal Raman microscope (excitation wavelength: 785 nm). (Reprinted with permission from [34]).

The human body can regenerate and repair itself after certain injuries, and for injuries such as tissue damage or organ failure, stem cell therapy is shown to accelerate regeneration. This repair mechanism occurs at the cellular and molecular level. Despite the standard medical treatments, drug-based therapies, and ongoing research on cardiovascular diseases, the clinical impact on society in terms of morbidity, mortality, and quality of life is still not understood [58]. With this motivation, a research group used confocal Raman spectroscopy to study the cytology of cells. They cultured and imaged human-induced hiPSC-derived cardiomyocytes (CM_hiPSCs_), pluripotent stem cells (hiPSCs), and adult rat ventricular cardiomyocytes (rCM_adult_) to understand their 3D morphology, cellular behavior, and distinct biochemical composition. The comparison points included (i) hiPSCs versus CM_hiPSC_ to check the degree of maturation at each step, and (ii) CM_hiPSC_ versus rCM_adult_ to understand tissue organization and alignment. The intensities of specific Raman peaks were volumetrically reassembled using computational tools by mapping the spatial resolution to highlight the cells’ main biochemical features and construct a visual 3D shape, as shown in Figure 6a (left). The 3D constructed morphology of hiPSCs matched the hiPSC colonies reported in the literature. CM_hiPSC_ was ~3 µm in height, while rCM_adult_ showed binucleated mature cells with elongated rod-like shapes and sarcomeres, as reported previously (Figure 6a). Four main biochemical features were evaluated: cytoplasm, nucleus, lipid, and glycogen. The phenylalanine peak at 1008 cm^−1^ assigned for protein content resembled the cell cytoplasm (highlighted in blue); the O–P–O stretch peak at 789 cm^−1^ corresponded to DNA, i.e., the nucleus (highlighted in red); the CH_2_ stretch peak at 2857 cm^−1^ corresponded to lipids (green); and the 485 cm^−1^ peak corresponded to glycogen (white). This comprehensive study comprising cell proliferation, differentiation, and maturation provided valuable information on physiology that can be applied in several fields such as developmental biology, tissue engineering, and regenerative medicine for improved clinical therapies [33].

In another study [33], scientists presented a label-free quantitative volumetric Raman imaging (qVRI) approach for cardiac stem cells. They assembled a confocal Raman spectroscopy setup and collected univariate imaging of distinct vibrational modes for the cells. The 3D morphology was volumetrically reconstructed by highlighting the Raman peaks specific to the cells’ biochemical components. The computational tools helped to identify and assign specific biomolecules based on the spatial resolution and create 3D Raman imaging datasets that could ultimately allow us to spatially monitor complex biological progressions such as cell differentiation and vascularization in 3D cell setups (Figure 6b) [33].

In a similar study [34], a Raman spectrometer was combined with a standard upright confocal microscope, but to identify changes in a single cell from either a well plate or fixed cells on standard glass slides (Figure 6c). In principle, when a laser beam focuses on a cell through a microscope lens, it gives higher resolution than a traditional stand-alone Raman spectrometer. Figure 6c shows the Raman spectrometer coupled to the charge-coupled device (CCD) of the camera to visualize, image, map, and collect the Raman spectra of the cells. Advantages of confocal Raman spectroscopy include raster-scan to collect full Raman spectra sequentially from each location, and mapping cells to generate a pseudo-colored map based on the composition of the cells [34].

**Figure 7:**
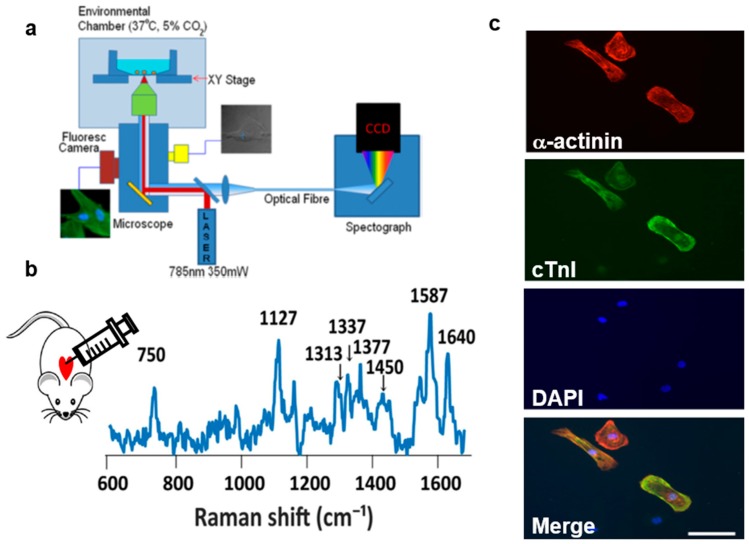
Raman imaging of cardiac cell. (**a**) Standard configuration of an inverted confocal Raman microscope (excitation wavelength: 785 nm). (Reprinted with permission from [55]). (**b**) Label-free acquisition of Raman spectra of a perfused rat heart under global ischemic conditions (excitation wavelength: 532 nm). (Reprinted with permission from [38]). (**c**) Evaluation of cardiomyocyte differentiation efficiency by immunofluorescence staining of beating embryoid bodies with α-actinin and cTnI (excitation wavelength: 785 nm). (Reprinted with permission from [36]).

A Raman spectrometer has also been combined with an inverted optical microscope, particularly for time-course cell imaging (Figure 7a). The advantage of this assembly is that cells can be cultured in standard cell chambers based on the dimension of the microscope stage while efficient collection of the Raman spectra from the bottom takes place. The inverted microscope was equipped with an environmental enclosure, thereby maintaining live cells at 37 °C with 5% CO_2_ atmosphere [55].

Impairment of blood vessels leads to a decrease in functional cardiomyocytes, resulting in a shortage of oxygen supply needed for cellular metabolism and eventually in myocardial ischemia [59,60]. Ischemic conditions lead to loss of mitochondrial membranes and increments of reduced cytochromes [61]. Cell biologists have studied myocardium viability by staining the mitochondrial membrane but were unable to identify the extent of myocardial ischemia, mainly during early, reversible situations [62]. With the progression of Raman microscopy, a group of researchers reported on label-free evaluation in the early ischemic phase of myocardial ischemia. The Raman spectra (excitation = 532 nm) in Figure 7b were acquired from the subepicardial myocardium tissue of a Langendorff-perfused rat heart. Figure 7b (right) displays two strong bands at 1587 cm^−1^ (reduced form of cytochrome c) and 1640 cm^−1^ (reduced form of cytochrome b) and two weaker ones at 750 cm^−1^ (reduced form of cytochrome c) and 1127 cm^−1^ (reduced form of cytochrome b). There are also other peaks observed at 1313 cm^−1^ (cytochrome c) and 1337 cm^−1^ (cytochrome b). In order to understand and correlate the Raman peaks from an early ischemic myocardium with other ischemic conditions, the rat heart was induced with both global ischemia (GI) and ischemic preconditioning (IPC). The Raman peaks remained the same, with an increase in peak intensity in the case of GI and a decrease in the case of IPC [38].

In stem cell therapy, cell fate is dependent on the source of host cells. Clinically, the treatment of a diseased myocardium involves applying a huge population of cardiomyocytes at the site of the infarct. Since the fate of these cardiomyocytes is dependent on the source cells, knowledge of cell physiology at different time points is essential. Raman microspectroscopy (RMS) has been used to understand the live-cell behavior of hESCs differentiated into cardiomyocytes in vitro. Time-resolved Raman spectra were recorded (from several hours) to detect changes at the molecular level. Immunofluorescence staining of cardiomyocyte differentiated embryoid bodies (EBs) displayed α-actinin and cTnI, which are crucial for contractile functions, as shown in Figure 7c. These beating EBs displayed Raman bands at 482, 577, 858, 937, 1083, and 1340 cm^−1^. Peaks at 482 and 577 cm^−1^ resembled cardiomyocyte-rich regions of the beating EBs. Integrating spectroscopic imaging for quality assessment until the end product differentiated cells will allow for effective clinical-based cell therapies [36].

#### 2.2.3. Raman Spectroscopy for Cardiac Tissues

**Figure 8:**
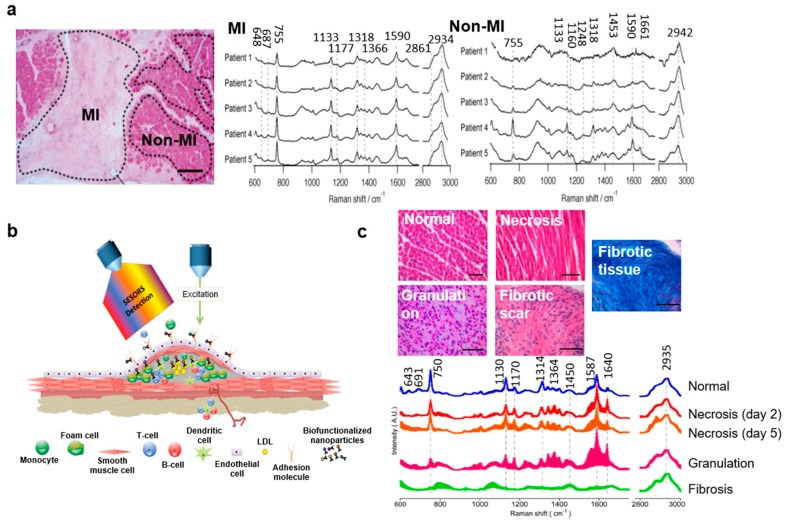
Raman imaging of cardiac tissues. (**a**) Label-free acquisition of Raman spectra of infarcted and noninfarcted ventricular myocardium excised from five patients (excitation wavelength: 532 nm). (Reprinted with permission from [39]). (**b**) SERS-based imaging for diagnosis of atherosclerosis. (Reprinted with permission from [10]). (**c**) Hematoxylin and eosin (H&E) stained normal, necrotic, and granulation tissue and fibrotic scar, and Azan stained fibrotic tissue (top), and the corresponding representative Raman spectra (bottom) (excitation wavelength: 532 nm). (Reprinted with permission from [40]).

Recently, researchers evaluated the viability of ischemic myocardial tissue by label-free Raman spectroscopy in patients undergoing cardiac surgery. Figure 8a (left) shows hematoxylin and eosin (H&E) stained myocardium with both the infarcted region (MI) (light pink) and noninfarcted (non-MI) (dark pink) tissue. Figure 8a (middle and right) shows the representative Raman spectra obtained from the five patients. The four signature peaks of cardiomyocytes at 755, 1133, 1318, and 1590 cm^−1^ corresponding to heme proteins were exhibited both by MI and non-MI. The non-MI tissues had higher peak intensities, as they consisted mostly of cardiomyocytes. Peaks at 1248, 1453, 1661, and 2942 cm^−1^ were from collagen, suggesting the presence of fibrosis in the MI tissue. The 755 cm^−1^ band corresponded to cytochrome c, cytochrome b5, myoglobin, and hemoglobin, and hence was found in both MI and non-MI. Peaks at 687, 1177, and 1366 cm^−1^ are specific to non-MI tissue. The results showed that the Raman peaks were consistent for all five patients, as shown in Figure 8a (middle and right). Due to high signal-to-noise ratio, Raman bands in close proximity are difficult to identify. In this regard, researchers have employed various statistical analysis models, such as multivariate spectral analysis, to identify specific positions of the Raman peaks. In order to distinguish the infarcted from the noninfarcted myocardial tissue, the researchers derived a prediction model using partial least squares regression–discriminant analysis (PLS-DA) in the Raman spectrum data [39].

In another recent study, Raman spectroscopy imaging was used for the diagnosis and identification of plaques, intended to understand the atherosclerotic condition. As shown in Figure 8b, the assembled surface enhanced Raman spectroscopy (SERS) setup could be applied for both ex vivo (for example, human vasculature) and in vivo (for example, mice) models. The researchers exploited the preresonance Raman effect to examine the molecular signatures inside blocked arteries caused by plaque buildup and find the inflammatory markers responsible for the manifestation of rupture-thrombosis or MI (commonly known as stroke or heart attack). As shown earlier, in resonance Raman (Figure 8c) the excitation wavelength overlaps the excited electronic state, resulting in an increase of scattering intensities by factors of 10^2^ to 10^6^, capable of reaching detection limits up to the 10^−9^ to 10^−12^ M range. For in vivo study, the researchers utilized a protein, intercellular adhesion molecule-1 (ICAM1), attached to gold nanoparticles (NPs) to detect ICAM-1 expression in aortic sinus tissues obtained from atherosclerotic-prone apolipoprotein E–deficient (apoe^−/−^) mice [10]. In a recent paper, Molly M. Stevens’s group at Imperial College London used Raman spectroscopy to study cardiovascular calcification [46]. The study revealed that the concentrations of apatite, triglyceride, and cholesterol increased and the concentration of β-carotene decreased in atherosclerotic plaque.

In another study, a multivariate discrimination model was used to create 2D images of different stages of MI from the Raman spectral signatures using PLS-DA. MI was created in 8-week-old female Wistar rats and Raman spectra were acquired at five sequential stages of the MI: normal tissue, necrosis (day 2), granulation tissue (day 5), fibrotic scar, and fibrotic tissue. Figure 8c (top) shows an image of H&E stained tissue from normal heart indicating healthy cardiomyocytes. After 24 h, i.e., the necrotic stage, there was an increase in eosinophil population (white bloods cells recruited to protect from damage), loss of cross-striations, and nucleus fragmentation. By days 4 to 7, invasion of macrophages started toward the infarcted tissue and granulation tissue was formed. By week 3, fibrosis of the infarcted tissue was observed. Peaks at 643, 691, 750, 1130, 1314, and 1587 cm^−1^ were assigned to normal tissue (as labeled in Figure 8c, bottom). Peaks at 643, 691, and 1314 cm^−1^ were for cytochrome c. Raman bands at 750, 1130, and 1587 cm^−1^ indicate cytochromes c and b5 in their reduced form. Necrotic tissues did not display reduced cytochrome c peaks, although they had weaker intensity peaks at 750 and 1314 cm^−1^. The intensity of peaks at 750 cm^−1^ was comparatively lower, indicating granulated tissue. The peaks at 1306 cm^−1^ and 1314 cm^−1^ were generally assigned to hemoglobin and cytochrome c, respectively. The CH_3_ stretching mode observed at 2941 cm^−1^ (shifted from 2935 cm^−1^, as observed in other stages) was due to fibrosis of the tissue and shows mature collagen. Since this technique involved label-free analysis of nonfixed tissues, future studies could be applicable for both in vivo acquisition and open heart surgery [40].

#### 2.2.4. Raman Spectroscopy for Whole Heart (Organ)

**Figure 9:**
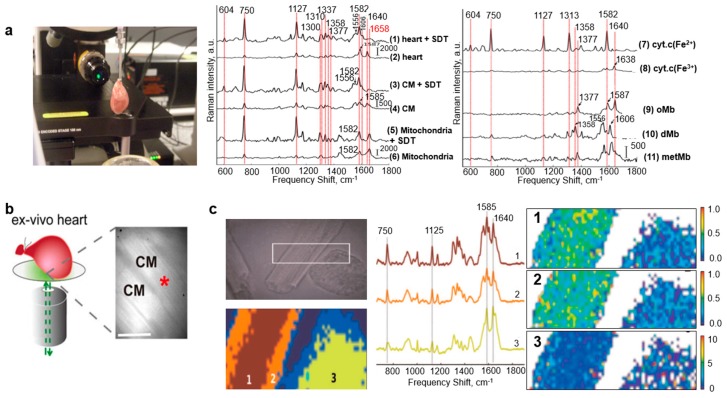
Raman imaging of whole heart. (**a**) Evaluation of an isolated rat heart using Raman microspectroscopy (top), acquisition of Raman spectra as per labels (middle and right) (excitation wavelength: 532 nm). (Reprinted with permission from [47]). (**b**) Schematic representation of the excised whole heart and optical setup of the slit-scanning apparatus (excitation wavelength: 532 nm). (Reprinted with permission from [48]). (**c**) Brightfield image of rod- and round-shaped cardiomyocytes (top left), representative Raman map (top right), Raman spectra of the highlighted area (bottom left), and Raman cluster analysis (bottom right) (excitation wavelength: 532 nm). (Reprinted with permission from [37]).

Raman spectroscopy has also been applied to study the reduction state of mitochondrial cytochromes and the extent of myoglobin oxygenation at the infarct site of whole rat heart. Myoglobin is a respiratory protein that supplies oxygen required for metabolic processes to mitochondria, which serve as the oxygen storehouse for mitochondrial cytochrome c oxidase. During hypoxia, the concentration of myoglobin spikes considerably, increasing the risk of a stroke. Figure 9a (left) shows a schematic representation of the setup and the position of the heart with respect to the objective and the laser used to study the relationship of hypoxia with myoglobin and ischemia. Figure 9a (middle) shows Raman spectra of perfused heart after reduction with sodium dithionite (SDT), perfused heart, CM mitochondria after reduction with SDT, CM, isolated CM mitochondria in partially oxidized state, and mitochondria. Figure 9a (right) shows Raman spectra of reduced cytochrome c (Fe2+), oxidized cytochrome c (Fe3+), oxymyoglobin (oMb), deoxymyoglobin (dMb), and metmyoglobin (metMb). Peaks at 750, 1127, 1587, and 1640 cm^−1^ were observed for perfused heart and after reduction with SDT. Low peaks at 1300, 1310, 1337, and 1377 cm^−1^ were observed in oxidized conditions. The addition of SDT generated reduced forms of cytochromes, increasing the intensity of peaks at the above-mentioned positions. Peaks at 750 and 1127 cm^−1^ corresponded to cytochrome c and b, respectively. Peaks at low myoglobin concentration, 1377, 1587, and 1640 cm^−1^, arose due to heme vibrations in oMb. Upon addition of SDT, deoxygenation occurred and caused a transition of oMb to dMb, generating low peaks at 1358, 1556, and 1606 cm^−1^ [47]. These myoglobins are intracellular scavengers of nitric oxide (NO) that help regulate its level in cardiac and skeletal muscles and regulates the mitochondrial respiration to prevent myocardial hypoxia or ischemia. The nitrite reductase activity of myoglobin leads to the production of NO in cells under hypoxia and results in inhibition of mitochondrial respiration. Several studies were performed to understand the intracellular redox state of myoglobin and cytochrome c (or the heme complex). Researchers preconditioned ex vivo cardiomyocytes with drugs to induce NO release at the single-cell level and monitored it using Raman microspectroscopy [63]. They also monitored the reduced levels of cytochrome c from hypoxia-induced rat cardiomyocytes and reoxygenated those cells to understand the cellular response thereafter [35]. Another study on acquisition of spectra from whole heart was conducted by assembling a Raman confocal microscope integrated with a slit-scanning apparatus, as shown in Figure 9b. The process of parallel detection and direct illumination reduced the acquisition time from hours to minutes and permitted precise tissue imaging [48].

Raman mapping and cluster analysis of live cardiomyocytes were also done by a group of experts to map the redox states of mitochondrial cytochromes. Figure 9c shows a brightfield image of the cardiomyocytes from which the Raman map was acquired. The color-coded maps reveal the concentration of different forms of cytochromes (such as c, c1, and b). The corresponding Raman peaks (750, 1125, and 1640 cm^−1^) are also shown in Figure 9c (bottom left). These three peaks were used for Raman spectral analysis by calculating the ratios of their peak intensities (I_750_:I_1640_, I_1125_:I_750_, and I_1125_:I_1640_), and to create pixel-by-pixel ratio-built maps. Label 1 corresponds to the reduced form of cytochromes c and c1, label 2 corresponds to the reduced form of cytochrome b, and label 3 corresponds to a higher ratio of the reduced form of cytochromes b and c in the periphery of the cell (Figure 9c, bottom right) [37]. Besides obtaining Raman maps from cells, ex vivo Raman maps have also been acquired from stenotic aortic valves to monitor mineral deposits and cholesterol and lipid levels [44,45].

## 3. Challenges and Future Perspective

The most significant property of Raman spectroscopy that makes it a suitable choice for clinical applications is its label-free nature. Furthermore, the capability of this technique to acquire near real-time measurements is another remarkable factor for clinical diagnosis of diseases. Accordingly, some handheld devices have been developed for in vivo study purposes [64]. However, the clinical applications of Raman spectroscopy have not become common yet, due to the inherent limitations of low signal for inelastic light scattering (Raman) compared to tissue autofluorescence, as well as the challenges that exist specifically for cardiovascular studies [38,65]. The best results for various biochemical applications can be achieved by striking a balance between acquisition time, spatial resolution, and spectral resolution. Providing such a balance has always been a challenge for clinical diagnostics. The goal of developing methods like SERS was mainly to increase the signal-to-noise ratio while decreasing the acquisition time [66]. Stimulated Raman scattering and anti-Stokes Raman scattering are other techniques that were aimed at reducing acquisition time while preserving ultra-high spatial resolution (~1 µm) by means of frequency multiplexing methods [67,68]. Meanwhile, translating these technologies into clinical use is considerably complex because of the large size of the instruments and difficulties in the efficient delivery and collection of photons. Moreover, miniaturization of instruments negatively affects the signal detection of Raman techniques during in vivo investigation. For ex vivo histopathology studies using Raman spectroscopy, preserving biological tissues in their native states is important, but difficult to ensure in practice. The best preservation can be achieved by keeping the sample in phosphate buffered saline (PBS) at 4 °C and using it within 3–4 h [69]. For long-term preservation, paraffin-fixed tissues can be employed. Meanwhile, it is difficult to confidently analyze the spectra of paraffin-fixed samples due to the considerable background peaks from paraffin. Ambient light interference is also an important consideration in nonendoscopic applications. Temporary elimination of ambient light during the Raman signal acquisition can considerably enhance detection. Spectrum normalization in accordance with the ambient background light can also mitigate this issue [70].

Although Raman spectroscopy is considered a promising tool for the diagnosis of cardiovascular diseases, there are still several challenges that need to be overcome before full utilization of this vibrational method can be achieved in clinical settings. One of the primary challenges in Raman spectroscopy of cardiac tissues is the limitation on the depth of penetration of the analysis due to strong scattering media of biological tissues. Therefore, collecting Raman signals from a buried region under more than a couple of millimeters of tissue is a difficult engineering task. Time-gated techniques can mitigate this problem to some extent. However, considerably more research is required on analyzing deeper tissues using Raman spectroscopy. Universal multiple-angle Raman spectroscopy (UMARS) [71] is another novel method, with Raman signals gathered from any geometry and angle to maximize the photon collection. The second most important challenge in this field is the complexity of acquired spectrum analysis from biological tissues because of the similarity of different vibrational modes in the spectra of lipids, proteins, cells, and bacteria. On the other hand, the complexity of biochemical spectra demonstrates the remarkable potential of this approach for biochemical applications by providing significant chemical specificity. Meanwhile, there is a lack of literature covering peak assignment for cardiovascular diseases. Moreover, correlation of the acquired peaks with the current literature is always necessary to make an unbiased judgment of the results. Raman imaging is another useful application of Raman spectroscopy, which is mainly restricted by the lack of adequate technological advancements in Raman microscopes capable of performing whole-animal imaging. This technique is considerably time-consuming and its application is limited to investigations of small areas (<1 mm × 1 mm) [72]. However, this limitation is more a technological than a scientific issue. Therefore, it is expected that much more powerful Raman microscopes will be introduced to the market in the near future.

Despite the aforementioned challenges and difficulties, Raman spectroscopy is a versatile, noninvasive, nondestructive, and highly chemical-specific method that has huge potential in cardiovascular disease studies. Continued advancements have been made to overcome the challenges and improve the instrumentation and data analysis in this approach. This review provides a glance at the potential of Raman spectroscopy for cardiovascular diseases. Principles, instruments, and applications of this method are discussed by elaborating the recent advancement in this realm. Despite numerous studies in the biochemical applications of Raman spectroscopy, cardiovascular Raman research is still considered to be a novel field that requires further studies.

## Figures and Tables

**Table 1:** Use of Raman spectroscopy in cardiovascular diseases. cTnI, cardiac troponin I; AgNP, silver nanoparticle; LSPR, localized surface plasmon resonance; CK-MB, creatine kinase–muscle/brain; LFA, lateral flow assay; LOD, limit of detection; NPT/ITO, nano-Pinetree array/indium tin oxide; hiPSC, human induced pluripotent stem cell; SERRS, surface enhanced resonance Raman scattering.

Category	Types	Findings	Reference
Biomarkers	cTnI	Detection of cTnI molecules after 3–4 h of stroke with ~1.3 ng/mL concentration. cTnI is adsorbed onto AgNPs to generate LSPR enhanced Raman signals.	[31]
	Myoglobin, cTnI, and CK-MB	LFA on paper microfluidics by immobilizing NPs encapsulated with Raman dyes. LOD for myoglobin was 50 ng/mL, cTnI and CK-MB were 10 ng/mL.	[32]
	Myoglobin	SERS-based myoglobin sensor based on Ag NPT/ITO substrate. LOD was 10 ng/mL.	[23]
Cardiac cells	➢ hiPSC-derived cardiomyocytes (CM_hiPSCs_) ➢ Adult rat ventricular cardiomyocytes (rCM_adult_)	Confocal Raman spectroscopy was used to study cell cytology. CM_hiPSCs_ displayed cardiomyocyte-like colonies. rCM_adult_ displayed elongated rod-like shapes and sarcomeres.	[33]
	Cardiomyocytes from rat	Raman spectrometer coupled with a charge-coupled device (CCD) of the camera was used to visualize, image, map, and collect the Raman spectra of the cells.	[34]
		Raman microspectroscopy (RMS) was used to evaluate NO release at the single-cell level.	[35]
	hESCs differentiated into cardiomyocytes	Raman microspectroscopy was used to study the fate of cardiomyocytes and acquire spectra from the beating embryoid bodies.	[36]
	Cardiomyocytes	Raman microspectroscopy was used to identify redox mitochondrial states and create a map to distinguish between rod- and round-shaped cardiomyocytes.	[37]
Tissues	Subepicardial myocardial tissue	Raman microscopy was used for label-free evaluation of mitochondrial membrane and reduced cytochromes in early myocardial ischemic phase.	[38]
	Ischemic myocardial tissue	Label-free Raman spectroscopy was used to study infarcted and noninfarcted regions from five patients who suffered a stroke.	[39]
	Myocardium infarcted tissue	Spontaneous Raman spectroscopy was used to identify the five sequential stages of myocardial infarcted tissue.	[40]
In vivo	Atherosclerosis	SERRS was used to study aortic sinus tissues by tagging with intercellular adhesion molecule-1 (ICAM1) protein attached to gold nanoparticles.	[10]
Ex vivo	Atherosclerosis	Spontaneous and coherent anti-Stokes Raman scattering (CARS) was used to study healthy and diseased tissues from biopsies of human gastrocnemius peripheral arterial disease (PAD) and control groups.	[41]
		Near-infrared Raman spectroscopy was used to evaluate lipid (cholesterol) and calcium salt content in human peripheral arteries.	[42]
		Raman spectroscopy was used to acquire spectra from skeletal muscle of PAD versus control.	[43]
		Raman spectroscopy was used to study stenotic aortic valves to monitor mineral deposits, and cholesterol and lipid levels.	[44,45]
		SERS was used to identify plaques in blocked arteries.	[10]
		Raman spectroscopy was used to study cardiovascular calcification.	[46]
	Whole heart	Raman spectroscopy was used to study the reduction state of mitochondrial cytochromes and myoglobin oxygenation at infarct sites of whole rat hearts.	[47]
		Raman confocal microscope integrated with a slit-scanning apparatus was used to acquire spectra from whole rat hearts.	[48]

**Table 2:** Suggested testing schedule for cardiac markers.

Marker	<6 h	6–12 h	12–24 h	24–48 h	>48 h
Myoglobin	+ + +	+	-	-	-
Troponin I	+	+ +	+ + +	+ + +	+ + +
Troponin T	+	+ +	+ + +	+ + +	+ + +
CK-MB	+	+ +	+ + +	-	-
MB-isoforms	+ +	+ + +	+	-	-
